# The beauty and complexity of the small heat shock proteins: a report on the proceedings of the fourth workshop on small heat shock proteins

**DOI:** 10.1007/s12192-023-01360-x

**Published:** 2023-07-18

**Authors:** Heath Ecroyd, Britta Bartelt-Kirbach, Anat Ben-Zvi, Raffaella Bonavita, Yevheniia Bushman, Elena Casarotto, Ciro Cecconi, Wilson Chun Yu Lau, Jonathan D. Hibshman, Joep Joosten, Virginia Kimonis, Rachel Klevit, Krzysztof Liberek, Kathryn A. McMenimen, Tsukumi Miwa, Axel Mogk, Daniele Montepietra, Carsten Peters, Maria Teresa Rocchetti, Dominik Saman, Angela Sisto, Valentina Secco, Annika Strauch, Hideki Taguchi, Morgan Tanguay, Barbara Tedesco, Melinda E. Toth, Zihao Wang, Justin L. P. Benesch, Serena Carra

**Affiliations:** 1 Molecular Horizons and School of Chemistry and Molecular Bioscience, University of Wollongong, Wollongong, NSW Australia; 2 Institute of Anatomy and Cell Biology, University of Ulm, 89081 Ulm, Germany; 3 Department of Life Sciences, Ben-Gurion University of the Negev, Beer Sheva, Israel; 4 Department of Molecular Medicine and Medical Biotechnologies, University of Naples “Federico II”, 80131 Naples, Italy; 5 Center for Protein Assemblies and Department Chemie, Technische Universität München, München, Germany; 6 Dipartimento di Scienze Farmacologiche e Biomolecolari “Rodolfo Paoletti” (DiSFeB), Dipartimento di Eccellenza, Università degli Studi di Milano, Milan, Italy; 7 Department of Physics, Informatics and Mathematics, University of Modena and Reggio Emilia, Modena, Italy; 8 Istituto Nanoscienze–CNR-NANO, Center S3, Modena, Italy; 9 State Key Laboratory of Chemical Biology and Drug Discovery, Department of Applied Biology and Chemical Technology, The Hong Kong Polytechnic University, Hung Hom, Hong Kong China; 10 Biology Department, University of North Carolina at Chapel Hill, Chapel Hill, NC 27599 USA; 11 Department of Synthetic Organic Chemistry, Institute for Molecules and Materials, Radboud University, Nijmegen, The Netherlands; 12 Department of Biomolecular Chemistry, Institute for Molecules and Materials, Radboud University, Nijmegen, The Netherlands; 13 Division of Genetics and Genomic Medicine, Department of Pediatrics, University of California - Irvine, Orange, CA 92868 USA; 14 Department of Neurology and Department of Pathology, University of California, Irvine, CA 92697 USA; 15 Department of Biochemistry, University of Washington School of Medicine, Seattle, WA 98195 USA; 16 Intercollegiate Faculty of Biotechnology, University of Gdansk, Gdansk, Poland; 17 Program in Biochemistry and Department of Chemistry, Mount Holyoke College, South Hadley, MA 01075 USA; 18 Cell Biology Center, Institute of Innovative Research, Tokyo Institute of Technology, Midori-ku, Yokohama, 226-8503 Japan; 19 Center for Molecular Biology of Heidelberg University (ZMBH), DKFZ-ZMBH Alliance, Im Neuenheimer Feld, 282 Heidelberg, Germany; 20 Department of Department of Chemical, Life and Environmental sustainability sciences, University of Parma, Parma, Italy; 21 Department of Clinical and Experimental Medicine, University of Foggia, 71122 Foggio, Italy; 22 Department of Chemistry, Dorothy Crowfoot Hodgkin Building, University of Oxford, Oxford, UK; 23 Kavli Institute for Nanoscience Discovery, University of Oxford, Oxford, UK; 24 Peripheral Neuropathy Research Group, Department of Biomedical Sciences and Institute Born Bunge, University of Antwerp, Antwerpen, Belgium; 25 Department of Biomedical, Metabolic, and Neural Sciences, University of Modena and Reggio Emilia, Modena, Italy; 26 Institute of Biochemistry, Biological Research Centre, Eötvös Loránd Research Network, Temesvári krt. 62, Szeged, H-6726 Hungary

**Keywords:** Small heat shock proteins, Molecular chaperones, Protein folding, Protein aggregation, Cellular stress, Protein homeostasis

## Abstract

The Fourth Cell Stress Society International workshop on small heat shock proteins (sHSPs), a follow-up to successful workshops held in 2014, 2016 and 2018, took place as a virtual meeting on the 17-18 November 2022. The meeting was designed to provide an opportunity for those working on sHSPs to reconnect and discuss their latest work. The diversity of research in the sHSP field is reflected in the breadth of topics covered in the talks presented at this meeting. Here we summarise the presentations at this meeting and provide some perspectives on exciting future topics to be addressed in the field.

## Introduction

Proteins are involved in virtually every biological process. In order to be functional, most proteins must adopt and maintain their native folded conformation. When these proteins do not remain folded, a process exacerbated by periods of cellular stress, they become non-functional and potentially misfold and even aggregate. The first line of defence against protein misfolding and aggregation are molecular chaperones. These consist of the ATP-independent *holdases*—which include the intracellular small heat shock proteins (sHSPs)—and the ATP-dependent *foldases*, such as HSP40/HSP70 and HSP90 members. The co-ordinated activities of molecular chaperones ensure proteins remain in their folded (functional) conformations. Under stress conditions, i.e. conditions which promote protein misfolding and aggregation, the level of chaperones increases significantly reaching up to 5% of the total cellular protein pool. Whilst much research has been done to characterise the mechanism of action of the ATP-dependent foldase chaperones, e.g. HSP40/HSP70, HSP90, and HSP60, the sHSPs have received comparatively little attention.

sHSPs are present in all kingdoms of life: in verterbrates, expression of sHSP isoforms varies with age, tissue, and levels of stress. Whilst sHSPs are often thought of as “generalist” molecular chaperones, this description does not fully capture their physiological roles in cells. For example, of the 10 human HSPBs, less than half show upregulated expression following heat stress, and HSPBs interact with various cellular components under normal physiological conditions (Kampinga et al. 2015). Even when considering the molecular chaperone function of sHSPS, the term *holdase* does not fully describe the mechanisms by which it interacts with client proteins (Ecroyd 2015, Ungelenk et al. 2016). In order to share and discuss these various aspects of sHSP structure and function, and as a mechanism to bring together investigators studying sHSPs, a workshop format was first proposed by Robert M Tanguay (Université Laval, Québec, Canada), a pioneer in the study of sHSP in *Drosophila melanogaster*.

Previous Cell Stress Society International (CSSI)-sponsored workshops focused on sHSPs have been held in Québec, Canada (2014, 2019) (Tanguay and Hightower 2015, Carra et al. 2019) and Bertinoro, Italy (2016) (Carra et al. 2017). These three workshops were held as face-to-face meetings; however, in 2022, the Fourth Cell Stress Society International workshop on sHSPs was held as a virtual meeting due to the ongoing challenges around travel at the time. Whilst the move to an online format restricted the opportunity for people to reconnect with colleagues and to establish connections with new ones, it did provide the chance for more researchers working in the sHSP field to participate in the meeting, including early career researchers and students. In fact, the response to this meeting was overwhelming—there were more than 95 people from around the world that registered to attend and up to 85 people in the presentation sessions held over the 2 days.

This fourth sHSP workshop was organised by Heath Ecroyd (University of Wollongong, Australia), Serena Carra (University of Modena and Reggio Emilia, Italy), and Justin Benesch (University of Oxford, UK) and was again generously supported by the CSSI. Workshop sessions were structured such that presentations from students and early career researchers were encouraged, which resulted in an excellent mix of students, early career researchers and group leaders presenting their work. Here we provide a summary of the work presented by speakers at the workshop, which together highlights *the beauty and complexity* of the sHSPs. The topics of the presentations ranged from structural studies aimed at understanding the molecular basis of sHSP oligomerisation and their interaction with client proteins, to functional studies aimed at uncovering the mechanism of action of sHSPs at the molecular level, to studies aimed at elucidating the role of sHSPs in human diseases. Based on these findings, some exciting future directions for the field were proposed and are summarised here.

## Structural studies of sHSPs and their interaction with client proteins

The sHSPs typically associate into oligomeric species which undergo dynamic (fast) subunit exchange. As an example, the archetypal mammalian sHSP, αB-crystallin (αBc, HSPB5), has a median mass of ~650 kDa and populates a range of large oligomers compromised of 20–50 monomers that are in dynamic equilibrium with smaller dissociated species (predominately monomers and dimers) (Hochberg and Benesch 2014). These attributes can make the study of the structure of sHSPs and the interactions with client proteins challenging. The presentations relating to the study of the structure of sHSPs highlighted how exploiting various recombinant forms of sHSPs and cutting-edge technologies can provide important new insights into how sHSPs assemble with each other and their client proteins.

The assembly state of HSP26 from *Saccharomyces cerevisiae* has intrigued the field some time, with 24mer and 40mer oligomeric states having been reported as dominant (Benesch et al. 2010). Recently, a new cryoEM study has shown that the HSP26 40mer forms large (C4 symmetric) oligomers (Muhlhofer et al. 2021). Like many sHSPs whose structures have been previously determined, the overall architecture can be viewed as a polyhedron composed of edges made up by sHSP dimers (Hilton et al. 2013): in this case a gyro-elongated square pyramid. Each subunit contains 9 potential phosphorylation sites, which form clusters both on the outside of the oligomer (mediated by the interlinked C-termini) and near the centre (mediated by the middle domain of the N-terminal region). Carsten Peters (Technical University of Munich, Germany) presented work showing that mimicking phosphorylation of HSP26 by introducing negative charges into these structural elements, leads to either unlocking of the otherwise buried N-terminal region or release of the interlocking C-terminal regions. In both cases, HSP26 becomes activated to bind client proteins. It is concluded from this work that phosphorylation allows *S. cerevisiae* to modulate HSP26 function independent of heat shock conditions.

Wilson Chun Yu Lau (The Hong Kong Polytechnic University, Hong Kong) described work aimed at elucidating the structural organisation of small heat shock protein (sHSP)-substrate complexes using state-of-the-art cryo-electron microscopy. The work focused on the stable complex formed between the *Arabidopsis thaliana* plastid sHSP, HSP21, and its natural substrate 1-deoxy-D-xylulose 5-phosphate synthase (DXPS) under heat stress (Yu et al. 2021). It was found that monomeric HSP21 binds across the dimer interface of DXPS and engages in multivalent interactions by recognising highly dynamic structural elements in DXPS. HSP21 partly unfolds its central alpha-crystallin domain to facilitate binding of DXPS, which preserves a native-like structure. This mode of interaction suggests a mechanism of sHSP anti-aggregation activity that may apply across a broad range of substrates, and emphasises that sub-oligomeric forms of sHSPs can play an important role in target recognition.

*Caenorhabditis elegans* encodes 16 different sHSPs, amongst them HSP17, which is evolutionarily distinct from other sHsps in the nematode. Annika Strauch (Technical University of Munich, Germany) reported that HSP17 has a unique expression pattern, structural organisation and chaperone function. Interestingly, HSP17 acts in a substrate-, concentration- and temperature-dependent manner as a holdase or an aggregase. Consistent with its presence under non-stress conditions, HSP17 is a permanently active chaperone interacting with hundreds of different substrate proteins under physiological conditions. The cryo-EM structure of HSP17 suggests that in the 24-mer complex, half of the N-terminal regions are involved in its chaperone function. These are located flexibly on the outside of the spherical oligomer, whereas the other half of the N-terminal regions is engaged in stabilising interactions in its interior. This allows the same region in HSP17 to perform different functions depending on the topological context (Strauch et al. 2023).

Rachel Klevit (University of Washington, USA) presented findings regarding the disordered N-terminal regions (NTRs) of sHSPs. Despite high similarity, HSPB4 and HSPB5 display remarkably different chaperone activity towards the lens protein, gammaD-crystallin, with HSPB5 being more active. Chimeric sHSPs revealed that chaperone activity follows the identity of the NTR. Finer parsing identified ten residues in the middle of the NTR that, when swapped between HSPB4 and HSPB5, recapitulate full NTR swaps. Klevit showed data from hydrogen-deuterium exchange followed by mass spectrometry, and UV-crosslinking experiments that demonstrate that the middle NTR region is an interaction hub in sHSP oligomers. This hub appears to govern the accessibility of the entire NTR to clients, and therefore the chaperone activity of sHSPs (Woods et al. 2023).

Unlike the canonical sHSPs, which typically form large oligomers, Zihao Wang (University of Oxford, UK) showed that cardiovascular heat shock protein (cvHSP, HSPB7) forms a weaker dimer than the other human sHSPs, such that it is predominantly monomeric in solution at physiological concentration. The crystal structure of cvHSP helps to explain why oligomerisation and dimerization are much less favoured compared to other sHSPs. Zihao Wang also presented data on the specific interaction between cvHSP and filamin C domain 24 (FLNCd24)—the binding of cvHSP results in a heterodimer, competing with FLNCd24 homodimerization. Phosphorylation of FLNCd24 at two different residues showed regulation of dimerization: either weakening the homodimer and strengthening the heterodimer or vice versa. This reveals a new mode of cytoskeletal regulation by a sHSP.

HSPB1 and HSPB5, the two most abundant human sHSPs, are thought to have originated in a gene duplication event about 400 million years ago. Dominik Saman (University of Oxford, UK) described investigations on how these two proteins co-assemble at the sub-unit level using native mass spectrometry, mass photometry and chemical kinetics modelling. He showed that these two proteins form large, heterogenous co-assemblies and seemingly still interact without bias for self- or co-assembly. Current work is focusing on studying the origins of this remarkably slow evolution, and Dominik hypothesises it may be due to functional constraints on the pair.

Enhanced molecular dynamics simulations and the Neural network algorithm EncoderMap (Lemke and Peter 2019) were combined to investigate the structure and dynamics of human HSPB8 and its neuropathological mutant K141E in the work presented by Daniele Montepietra (University of Parma, Italy). Given the absence of experimental structural characterisation, high-dimensional data sets of 3D structures were generated and projected into 2D maps from which the most significant conformations were extracted and interpreted. The findings suggest that the missense mutation increases the structural variability and compactness of HSPB8, whilst rearranging the exposed hydrophobic patches. These results offer the possibility of rationalising the effects of the pathogenic mutation in terms of its conformational changes (Montepietra et al. 2022).

Ciro Cecconi (University of Modena and Reggio Emilia, Italy) described work using optical tweezers to study the microscopic details of the mechanisms of action of human HSPB8 and its pathogenic mutant K141E. Using the maltose binding protein as a client, they showed that HSPB8 hinders protein aggregation without affecting the native folding process. This unique mechanism of action seemingly relies on the interaction of HSPB8 with misfolded aggregated species forming at the early stages of aggregation. The K141E mutation decreases the chaperone affinity for non-native species, thus impairing its anti-aggregation activity.

Together, these presentations highlighted the various modes of interactions involved in the assemblies sHSPs make with themselves and their client proteins and the role of phosphorylation in regulating them. It is exciting to see sHSP researchers exploiting techniques such as cryo-electron microscopy, native mass spectrometry and single-molecule microscopy to study these interactions. The dynamic and heterogenous nature of the interactions means that not only will these cutting-edge techniques help uncover new aspects to sHSP structure and function, it showcases the abilities of these techniques to be used for the study of dynamic protein complexes.

## Studying the function of sHSPs

As a consequence of their capacity to interact with and stabilise aggregation-prone client proteins through their molecular chaperone function, sHSPs play a role in numerous cellular pathways including cellular growth and differentiation, cytoskeletal function and apoptosis. Presentations during this sHSP workshop described work investigating roles for sHSPs in fields spanning the formation of bimolecular condensates through to the integrity of neuronal dendrites. Moreover, the presentations highlighted that sHSPs play vital roles across all phyla of life.

*Escherichia coli* has two small heat shock proteins, IbpA and IbpB. Tsukumi Miwa (Tokyo Institute of Technology, Japan) and colleagues recently reported that oligomeric IbpA self-represses the translation of IbpA and IbpB via the interaction with the 5′ untranslated region of the sHSP mRNAs (Miwa et al. 2021). Tsukumi Miwa explained that this self-regulation suppresses the expression of extra sHSPs in an aggregation-free environment. Oligomerized IbpA suppresses its translation via mRNA secondary structure, but its specificity was unclear. Work searching for novel regulatory targets of IbpA found that the translation of heat shock transcription factor σ32 is down-regulated by IbpA (Miwa and Taguchi 2023). These results reveal an unexplored role for IbpA in regulating the heat shock response at a translational level in *E. coli*.

Following on from Miwa’s presentation, Hideki Taguchi (Tokyo Institute of Technology, Japan) shared unpublished results on the difference between IbpA and IbpB, since IbpB does not have self-repression activity even though the two Ibps are highly similar. Systematic analyses revealed that a conserved residue in IbpA, R93, which is not present in IpbB, is solely responsible for this difference in activity between IbpA and IbpB.

Yevheniia Bushman (Technical University of Munich, Germany) reported on work investigating the *C. elegans* sHSP16s, namely HSP16.1, HSP16.2, HSP16.41 and HSP16.48. The pairwise sequence similarity—up to 93% between the family members—is reflected in structural and functional similarities. Chaperone activity assays show that the sHSP16s demonstrate features of aggregases or holdases. Subunit-exchange analysis shows that the sHSP16s are capable of hetero-oligomeric sub-unit exchange of oligomer subunits in vitro. Co-immunoprecipitation followed by mass spectrometry analysis identified not only a broad range of shared clients, but also binding partners that were specific for each sHSP. It was suggested that the existence of these homologous sHSP16s is advantageous as it allows the sHSPs to cover the broad range of destabilised clients present during cellular stress.

Krzysztof Liberek (University of Gdansk, Poland) described the use of ancestral reconstruction to study the evolution of sHSPs in *Enterobacterales*. This order of bacteria has either a two-component sHSP system (IbpA and IpbB), or a single sHSP (IpbA) that performs the functions of both. In the *Erwiniaceae* clade, the IbpB gene was lost, and the modern-day single sHSP evolved from an ancestral IbpA specialised in high-affinity client binding. In this sHSP, the residue substitutions Q66H and G109D, which localise to the β4-β8 groove of the alpha-crystallin domain, are key contributors to the functional differences between the ancestral strong client binder and the modern-day single sHSP. Biochemical analyses indicated that these substitutions weaken the interaction between the alpha-crystallin domain and aggregated clients, thus facilitating sHSP displacement from aggregates by the HSP70 system.

Maria Teresa Rocchetti (Università di Foggia, Italy) presented work establishing, for the first time, the chaperone and lipochaperone activity of three sHSP, i.e. sHSP1, sHSP2 and sHSP3, from a probiotic model organism, i.e. *Lactiplantibacillus plantarum*. The recombinant forms of these three sHSPs inhibited protein aggregation in vitro, in a concentration- and pH-dependent manner. Furthermore, the recombinant sHSPs exhibited a different holdase capacity during prolonged heat shock depending on whether they act alone or in combination. The highest holdase activity was found for the mixture of the three sHSPs suggesting they can act synergistically with regard to efficient trapping of heat-destabilised client proteins. The three sHSP modulated liposome membrane fluidity differently, with a clear lipochaperone activity for sHSP2. The different apparent functions of the three sHSPs of *L. plantarum* may account for the organism’s high adaptability and capacity to tolerate various stress conditions (Rocchetti et al. 2023).

The protective properties of sHSPs during temperature stress are well-documented. Jonathan Hibshman (University of North Carolina, USA) found that sHSPs can also serve a protective role during desiccation. Multiple sHSPs from tardigrades (*Hypsibius exemplaris*) or humans could improve bacterial desiccation survival. Further analysis of some tardigrade sHSPs revealed their capacity to form oligomers and ability to reduce heat-induced protein aggregation. Additionally, some tardigrade sHSPs limited desiccation-induced protein aggregation and protected against loss of enzyme function following desiccation—suggesting a biochemical function as a chaperone for dried proteins. These results indicate that sHSPs may be deployed as effective desicco-protectants (Hibshman et al. 2023).

In yeast, the sHSP HSP42 sequesters misfolded proteins into large inclusions (Specht et al. 2011). Other species do not encode for an HSP42 homologue raising the question whether sequestration represents an evolutionarily conserved sHSP activity. Axel Mogk (University of Heidelberg, Germany) reported on a screen for sHSP sequestrases that made use of a temperature-sensitive growth defect of yeast sequestrase mutants. Mogk’s team found that a subset of *Caenorhabditis elegans* sHSPs, including HSP16.1/2, restore growth and the sequestration of misfolded reporters. HSP16.2 is strongly upregulated in long-lived *C. elegans daf-2* mutants and contributes to longevity. Their findings demonstrate that sequestration represents an ancient and cytoprotective activity of the sHSP family (Shrivastava et al. 2022).

When it comes to studies on human sHSPs, Joep Joosten’s (Radboud University, The Netherlands) presentation demonstrated that human HSPB2 forms condensates through liquid-liquid phase separation in the nuclei of HEK293 cells. Expression of an equimolar amount of HSPB3 resulted in nuclear and cytoplasmic aggregation of HSPB2 and HSPB3, whereas co-expression of a moderate amount of HSPB3 resulted in a diffuse nuclear distribution of the two chaperones. Both liquid condensates and solid-like aggregates are reversible, as they can be dissolved by shifting the HSPB2:HSPB3 stoichiometry after their formation. Proximity labelling revealed that unbound nuclear proteins can freely shuttle in and out of HSPB2 condensates, whilst disordered proteins and autophagy factors get sequestered in, or are recruited to HSPB2:HSPB3 aggregates (Joosten et al. 2022).

It has been previously shown that human HSPB5 phosphorylated at serine residues 45 and 59 is neuroprotective by inhibiting dendritic rarefaction, a hallmark of neurodegenerative diseases (Bartelt-Kirbach et al. 2021). Britta Bartelt-Kirbach (University of Ulm, Germany) presented work aimed at identifying the molecular mechanism behind this finding. To do so, the team investigated the influence of phosphorylation on the binding of HSPB5 to the microtubular protein, tubulin, by pull-down assays. Whilst non-phosphorylatable HSPB5 had no effect, mutants mimicking phosphorylation at one site showed slightly better binding and pseudophosphorylation of two or three serine residues resulted in significantly stronger binding to tubulin than wild-type HSPB5. Overall, it was concluded that phosphorylation of HSPB5 increases its binding to tubulin and thereby protects microtubular and dendritic integrity.

A quantitative analysis of the basal chaperone system across human tissues revealed that the expression levels of most chaperones show tissue-specific changes, although they are ubiquitously expressed (Shemesh et al. 2021). Anat Ben-Zvi (Ben-Gurion University, Israel) reported on work showing that related human tissues have similar chaperone expression patterns that are conserved in evolution. However, of note, sHSPs organisation differs markedly from other chaperone families—sHSPs are more variably expressed, more tissue-selective, but less essential for growth than other chaperones (Shemesh et al. 2021). This organisation connects the chaperone system, specifically the sHSPs, to the cellular proteome folding requirements, suggesting that the varied expression of chaperones could determine the folding capacity of cells and tissues, and thus their susceptibility to protein misfolding diseases (Nisaa and Ben-Zvi 2022).

Morgan Tanguay (Mount Holyoke College, USA), an undergraduate student in the research group of Kathryn McMenimen, described a new method for evaluating multiplexed HSPs from mouse brain samples to enable understanding of multiple stress variables on chaperone protein expression. The team are developing a novel fluorescent immunoassay to quantify multiplexed HSP expression using fluorescent microbead (xMap) technology. HSP-specific (HSP27 or HSP70) fluorescent microbeads were constructed. Initial western blot analysis confirmed that HSP27- and HSP70-conjugated microbeads are specific to targeted HSPs. Further studies will be undertaken to test that HSPs in processed mouse brain samples are able to be appropriately visualised and quantified. Successful development of a multiplex immunoassay of HSPs will enable complex comparisons of HSPs during organismal stress response.

As with the presentation describing structural studies of sHSPs, a common theme across these reports was the use of innovative techniques and models to interrogate sHSP function by researchers within the sHSP field. Together, these studies highlight the importance of sHSPs in the normal functioning of cells and in conferring stress tolerance. In addition, these presentations highlighted that sHSP activity is regulated in multiple ways in the cell, including through changes in expression levels, localisation, formation of protein deposits and recruitment into biomolecular condensates, and post-translational modifications. Understanding the structure-function relationships that mediate changes in activity within the context of the cellular environment continues to be an area of intense interest.

## The role of sHSPs in human diseases

In order to fulfil their biological function as molecular chaperones, sHSPs must be able to bind to many different aggregation-prone proteins to stabilise them and prevent their further misfolding and aggregation. It is therefore not surprising that disruptions to this function, either due to mutations in sHSP themselves or their clients, is implicated in human pathologies as diverse as neurodegenerative diseases, myopathies, cataracts and cancers. Presentations at the workshop focussed on the role of sHSPs in neurodegenerative diseases, including the mutant forms of sHSPs that cause disease.

Mutations in *HSPB1* and *HSPB8* cause axonal Charcot-Marie-Tooth (CMT2) disease, distal hereditary motor neuropathy (distal HMN) or distal myopathy (Evgrafov et al. 2004, Irobi et al. 2004, Vendredy et al. 2020). Induced pluripotent stem cell (iPSC)-derived motor neurons carrying the *HSPB1*_P182L or *HSPB8*_K141N missense mutations recapitulates CMT2/distal HMN as defects in axonal transport and aberrant mitochondrial metabolism (Van Lent et al. 2021). Furthermore, the HSPB8^K141N/K141N^ knock-in mouse model shows defective motor performance, muscle weakness and alterations in the axonal structure. The iPSC-derived motor neurons show a reduction in the LC3-positive vesicles (Haidar et al. 2019), and accumulation of LC3IIB and SQSTM1/p62 is detected in the motor axons of the HSPB8 knock-in model (Bouhy et al. 2018), suggesting autophagy as a common defective mechanism in CMT2/distal HMN caused by sHSP mutations. Angela Sisto (University of Antwerp, Belgium) described work aimed at rescuing defective autophagy as a potential therapeutic strategy for CMT2/distal HMN. Mouse embryonic fibroblasts (MEF) isolated from the GFP-LC3/HSPB8^K141N/K141N^ mouse show a significant deficit in autophagosome formation. The MEF line was used to screen a library of annotated compounds to identify novel autophagy inducers that could stimulate autophagosome formation in the K141N mutant background. Selected hits were found to induce conversion of LC3BI to LC3BII, preserved the motor neuron neurite network during differentiation, promoted mitochondrial morphology remodelling and could consequently improve the overall neuronal homeostasis in the context of CMT2.

HSPB8 interacts with BAG3, HSP70 and STUB1 in participating in chaperone-assisted selective autophagy (CASA). Different neuromuscular disease-associated frameshift (fs) mutations in the *HSPB8* gene result in encoded HSPB8 proteins with a common aberrant C-terminal extension. Barbara Tedesco (University of Milan, Italy) presented work performed in collaboration with the research groups of Vincent Timmerman (University of Antwerp, Belgium) and Virginia Kimonis (University of California–Irvine, USA) showing that HSPB8_fs mutants are highly insoluble and tend to aggregate. As the HSPB8_fs mutants aggregate they sequester both wildtype HSPB8 and other members of the CASA complex. As a result, misfolded ubiquitinated substrates are trapped in HSPB8_fs aggregates, as well as autophagy receptors. Notably, the C-terminal extension of HSPB8_fs mutants is intrinsically aggregation-prone. It was concluded that this represents a novel gain-of-toxic-function mechanism through which different HSPB8_fs mutations cause neuromuscular diseases (Tedesco et al. 2023).

Amyotrophic lateral sclerosis and frontotemporal dementia are two neurodegenerative diseases characterised by the presence within neurons of toxic cytoplasmic inclusions containing an insoluble form of the TAR DNA-binding protein of 43 KDa (TDP-43) and its C-terminal fragments of 35 kDa and 25 kDa (TDP-35 and TDP-25, respectively) (Casarotto et al. 2022). Elena Casarotto (University of Milan, Italy) presented work aimed at evaluating a possible role for the CASA-complex in targeting TDP species to extracellular vesicles. An allosteric inhibitor of HSP70, JG-98, prevented CASA-complex formation and reduced the intracellular content of the TDP species, HSP70, BAG3 and HSPB8. In contrast, JG-98 treatment increased the amount of TDP species and HSPB8 in extracellular vesicles, but not of HSP70 or BAG3. It was concluded that HSPB8 may play a key role in the cellular secretion of various forms of TDP via extracellular vesicles, independent of its role in the formation of the CASA-complex.

A unique autosomal dominant rimmed vacuolar myopathy, caused mainly by frameshift mutations of the *HSPB8* gene, is associated with a distal myopathy that leads to progressive generalised weakness. Muscle biopsies show fatty replacement, fibrosis, rimmed vacuoles and aggregates that contain TDP-43. Virginia Kimonis (University of California–Irvine, USA) presented work in which they found that reduced HSPB8 expression increased TDP-43 and autophagy pathology in patient fibroblasts and myoblasts (Al-Tahan et al. 2019). A knock-in *Hspb*8 mouse model of the c.515dupC *Hspb*8 variant, which is seen in two families, showed, starting from 6 months, muscle weakness and muscle pathology similar to that seen in the human disease. The group are using different approaches, including novel drugs and gene therapy strategies, to address the gain of function—and possible loss of function—as the mechanism of HSPB8-associated myopathy.

To date, there is still no successful strategy to treat Huntington’s disease. Raffaella Bonavita (University of Naples, Italy) described a novel functional role for the HSPB1-p62/SQSTM1 complex, which acts as a cargo loading complex, allowing the unconventional secretion of mutant huntingtin (HTT) into extracellular vesicles. HSPB1 interacts preferentially with polyQ-expanded HTT compared with the wild type HTT protein, and affects its oligomerisation. Furthermore, levels of HSPB1 correlate with the rate of mutant HTT secretion in a manner which is controlled by the PI3K/AKT/mTOR signalling pathway. These HTT-containing vesicular structures are biologically active and able to be internalised by recipient cells (Bonavita et al. 2023). Thus, in this case, it appears that HSPB1 activity may be involved in facilitating the prion-like spreading of mutant HTT.

Parkinson’s disease is associated with α-synuclein aggregation, which can be triggered by lipid membranes. Intriguingly, sHSPs can interact with lipids and prevent α-synuclein aggregation. However, it remained to be established whether sHSPs prevent α-synuclein lipid-induced aggregation. The Vendruscolo lab developed a three-pronged approach that allows to disentangle the main three steps of α-synuclein aggregation: primary nucleation, fibril elongation and secondary nucleation (Galvagnion et al. 2015). Valentia Secco (University of Modena and Reggio Emilia, Italy) reported that, using this approach, their team has found that different sHSPs bind to α-synuclein monomers or fibrils and compete with α-synuclein for binding to lipids. Thus, sHSPs use multiple strategies to block α-synuclein aggregation, modulating the interplay between proteins and lipids. From this work, it can be concluded that sHSPs may be a therapeutic target in the context of Parkinson’s disease.

Melinda Toth (ELKH Biological Research Centre, Hungary) described work that has revealed a complex regulatory role of HSPB1 in inflammation and cell death induced by acute brain injury. Ethanol-induced neurodegeneration was accompanied by significantly higher expression of inflammatory cytokines and more robust glia activation in HSPB1 overexpressing transgenic mice than in their wild-type littermates. However, this more intense inflammation was not followed by increased levels of apoptosis, suggesting a compensatory anti-apoptotic function of HSPB1. Using primary cell cultures, it was found that, at least in part, intracellular HSPB1 is responsible for the regulatory effects (Dukay et al. 2021). The possible balancing role of HSPs in inflammation might make them a potential therapeutic target in inflammatory diseases.

It is clear from these presentations relating to the role of sHSPs in disease that strategies aimed at restoring (in the case of mutations to sHSPs) and/or boosting sHSP function may be beneficial in the context of particular disease states, such as some (but not all) neurodegenerative diseases. A challenge in doing so, however, will be the need to precisely target the cells and tissues involved in disease onset and progression. This is because increased levels of sHSPs are associated with some cancers.

## Summary and future directions for the sHSP field

Through their co-ordinated activities, ATP-dependent and ATP-independent molecular chaperones ensure the proteome remains folded and functional. Yet we still do not understand the fundamental mechanisms by which this chaperone machinery works. One reason for this gap in knowledge is that most research efforts have been undertaken to characterise the mechanism of action of the ATP-dependent chaperones, e.g. HSP40/HSP70, HSP90 and HSP60, whilst the ATP-independent chaperones, including the sHSPs, have received comparatively little attention. As a result, despite being an integral part of the system that sustains organismal protein homeostasis and maintains cell viability in the face of cellular stress, there is a critical gap in our understanding of how the components of the molecular chaperone machinery work together. This highlights the importance of workshops on sHSPs as a means of bringing together researchers working in the sHSP field. Moreover, it is through multidisciplinary approaches and the efforts of researchers to share their expertise and knowledge that we will be able to uncover the beauty and complexity of the sHSPs.
